# The Kv10.1 Channel: A Promising Target in Cancer

**DOI:** 10.3390/ijms23158458

**Published:** 2022-07-30

**Authors:** Enoch Luis, Arely Anaya-Hernández, Paulina León-Sánchez, María Luisa Durán-Pastén

**Affiliations:** 1 Cátedras CONACYT—Instituto de Fisiología Celular, Universidad Nacional Autónoma de México, Circuito Exterior s/n, C.U., Ciudad de México 04510, Mexico; 2 Laboratorio Nacional de Canalopatías, Instituto de Fisiología Celular, Universidad Nacional Autónoma de México, Circuito Exterior s/n, C.U., Ciudad de México 04510, Mexico; paulina_leon_s369@ciencias.unam.mx (P.L.-S.); mlduran@ifc.unam.mx (M.L.D.-P.); 3 Centro de Investigación en Genética y Ambiente, Universidad Autónoma de Tlaxcala, Km. 10.5 Autopista Tlaxcala-San Martín, Tlaxcala 90120, Mexico; arely.anayahernandez@uatx.mx

**Keywords:** oncogenic channel, Kv10.1, hallmarks of cancer, oncochannelopathies

## Abstract

Carcinogenesis is a multistage process involving the dysregulation of multiple genes, proteins, and pathways that make any normal cell acquire a cancer cell phenotype. Therefore, it is no surprise that numerous ion channels could be involved in this process. Since their discovery and subsequent cloning, ion channels have been established as therapeutic targets in excitable cell pathologies (e.g., cardiac arrhythmias or epilepsy); however, their involvement in non-excitable cell pathologies is relatively recent. Among all ion channels, the voltage-gated potassium channels Kv10.1 have been established as a promising target in cancer treatment due to their high expression in tumoral tissues compared to low levels in healthy tissues.

## 1. Introduction

Ion channels are pore-forming proteins that facilitate the movement of ions through the plasma membrane following their electrochemical gradients. Some ion channels are activated when they detect changes in the electrical potential across the membrane (voltage-gated), and others by extracellular or intracellular signals (ligand-gated) such as neurotransmitters or second messenger, respectively [1,2].

Voltage-gated ion channels (VGICs) are the main proteins that define cell excitability, and they are classified according to the ion moving through them (Na^+^, K^+^, Ca^2+^) [3]. In excitable cells (secretory cells, muscles, or neurons), VGICs generate regenerative responses called action potentials, which initiate major cellular events, including vesicular exocytosis, neurotransmitter release, or muscle contraction.

Around 145 VGIC have been described in mammalian cells, 40 of these are voltage-gated potassium channels (Kv), making them the most diverse (structurally and functionally) channel family [4]. Kv channels are expressed in excitable and non-excitable cells. Their activity regulates several biological processes, including control of neuronal excitability, heart rate, electrolytic balance, and cell division [4,5]. Given their wide localization, diverse structural arrays, and multiple physiological functions, Kv channel malfunctions are associated with several diseases and, consequently, are considered ideal drug discovery targets [6,7,8].

## 2. The Voltage-Gated Potassium Channel Kv10.1

According to their amino acid sequence homology, Kv channels are divided into 12 subfamilies (Kv1 to Kv12) and are assembled as tetramers. Kv10, Kv11, and Kv12 channel subtypes form the EAG (*ether-à-go-go)* family all regulate the K^+^ movement and work as modulators of cell excitability in different brain regions and heart [9,10]. The name EAG was assigned by a *Drosophila melanogaster* mutant fly, which exhibited a hyperexcitable phenotype characterized by leg-shaking under ether anesthesia [11]; later, cloning revealed that this phenotype was produced by the loss of a voltage-gated potassium channel, the Kv10.1 (also named eag1) [12].

### 2.1. The KCHN1 Gene

In humans, the Kv10.1 channel is encoded by the Potassium Voltage-Gated Channel Subfamily H Member 1 (*KCHN1*) gene. *KCNH1* is located in chromosome 1, band q32.2, and comprises 457,343 bases and 12 exons [13]. Homologous genes have also been identified in *Drosophila melanogaster*, chicken, cow, zebrafish, mouse, and rat [12,14,15,16,17,18]. Neurodevelopmental disorders associated with a gain-of-function of the *KCNH1* include the Temple-Baraitser Syndrome and Zimmermann-Laband Syndrome [19,20].

### 2.2. Kv10.1 Structure

In humans, the *KCNH1* gene encodes a 989 amino acid protein with an estimated molecular mass of 111,423 Da. Like other Kv, a functional Kv10.1 is formed by assembling four pore-forming subunits. The transmembrane core of each subunit consists of six transmembrane α-helices (S1–S6). Segments S1–S4 form the voltage sensor domain, with S4 carrying the positively charged amino acids that move in response to a depolarization of the membrane potential. Segments S5–S6 form the pore domain of the channel, and the loop P (among S5–S6) is responsible for the K^+^-selectivity. Kv10.1 has large intracellular N- and C-termini, which account for ~70% of its molecular mass; also, both intracellular termini have several modulatory domains. The N-terminus contains a Per-Arnt-Sim (PAS) domain. The C-terminus contains a cyclic nucleotide-binding homology domain (cNBHD), a C-linker, a ciliary localization signal, and a tetramerizing coiled-coil domain (TCC) [21,22]. Moreover, three calmodulin-binding domains (CaMBD) have been described in both termini. As well as other ion channels, Kv10.1 undergoes N-linked glycosylation (posttranslational modification) in the N388 and N406 sites, which is essential for the correct trafficking of the channels to the membrane [21,23].

Notably, at least four splice variants of the human *KCNH1* gene have been described. Two similar variants named Kv10.1a (the originally cloned) and Kv10.b (a long splice variant with additional 27 amino acids) form active ion channels with similar electrophysiological properties [15,24]. Additionally, two shorter non-functional channel variants (lacking their transmembrane segments) known as E65 and E70 (with a molecular weight of 65 and 70 kDa, respectively) have been detected in melanoma cell lines (IPC298 and IGR39), neuroblastoma cell lines, and the human brain [25].

### 2.3. General Biophysical and Pharmacological Properties

Kv10.1 channels are activated (open) at positive membrane potentials. One distinguishing property of Kv10.1 is that their activation-kinetic is strongly dependent on membrane potential: Kv10.1 currents activate faster when the membrane potential is more depolarized. In patch-clamp experiments, during a sustained depolarization stimulus, Kv10.1 activation is generated, slowly activating outward currents that do not show inactivation in the presence of the stimulus [21].

Due to the high homology with other members of the EAG family, finding a selective Kv10.1 blocker has been challenging. Several Kv10.1-blockers have been described [26,27]; however, none of these compounds are sufficiently specific for Kv10.1, having many targets. Classical Kv blockers, like TEA or 4-AP, show only a slightly inhibitory effect on Kv10.1 activity [12]. The small molecule and anti-histaminic drug astemizole is perhaps the best known and most used Kv10.1 blocker [28]. Recently, antibody engineering has emerged as a promising pharmacological tool for studying ion channels involved in diverse diseases, including cancer [29,30]. Currently, a specific monoclonal antibody (mAb56) and a nanobody coupled to TNF-related apoptosis-induced ligand (TRAIL) reduce cell growth and selectively induce apoptosis in cell lines expressing Kv10.1, respectively [31,32].

### 2.4. Tissue Expression

Kv10.1 channels are proteins embedded in the plasma membrane; however, it is also possible to find them in the membrane of subcellular structures: at the inner nuclear membrane [33], intracellular vesicles [34], and close to the primary cilium [35].

In healthy or physiological conditions, Kv10.1 is virtually undetected in peripheral tissues, only showing an enriched expression in specific brain regions (see the GTEx portal: www.gtexportal.org/home/gene/KCNH1, accessed on 24 July 2022). Through different approaches, either as protein or mRNA, Kv10.1 has been detected in the olfactory bulb, cerebral cortex, hippocampus, hypothalamus, striatum, and the presynaptic terminal of the parallel fiber-Purkinje cell synapses [10,13,24,36,37]. The study of the functional role of Kv10.1 in these brain regions in a physiological context remains incomplete. Kv10.1 is also transiently expressed during myoblast differentiation [13,38]. Kv10.1 expression is also reported in 100% of cervical samples of pregnant women, in contrast with a lower expression in non-pregnant ones, suggesting a hormonal regulation of the channel [39]. Moreover, Kv10.1 was reported in stem cells localized at colonic crypts [40].

**Figure 1:**
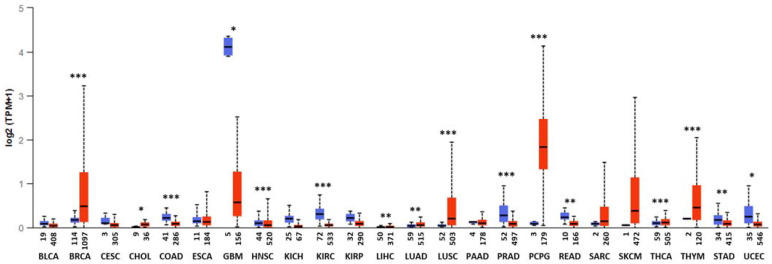
mRNA expression of *KCNH1* across TCGA cancers in normal and tumor samples. Log of transcript per million (TPM) of *KCNH1* mRNA from normal samples (blue box) and tumor samples (red box). The abscissa axis shows the number of samples and the type of tumor. The cancer genome atlas (TCGA), bladder urothelial carcinoma (BLCA), breast invasive carcinoma (BRCA), cervical squamous cell carcinoma (CESC), cholangiocarcinoma (CHOL), colon adenocarcinoma (COAD), esophageal carcinoma (ESCA), glioblastoma multiforme (GBM), head and neck squamous cell carcinoma (HNSC), kidney chromophobe (KICH), kidney renal clear cell carcinoma (KIRC), kidney renal papillary cell carcinoma (KIRP), liver hepatocellular carcinoma (LIHC), lung adenocarcinoma (LUAD), lung squamous cell carcinoma (LUSC), pancreatic adenocarcinoma (PAAD), prostate adenocarcinoma (PRAD), pheochromocytoma and paraganglioma (PCPG), rectum adenocarcinoma (READ), sarcoma (SARC), skin cutaneous melanoma (SKCM), thyroid carcinoma (THCA), thymoma (THYM), stomach adenocarcinoma (STAD), uterine corpus endometrial carcinoma (UCEC). * *p* < 0.05, ** *p* < 0.01, *** *p* < 0.0001. Data obtained from UALCAN (http://ualcan.path.uab.edu, accessed on 24 July 2022) [56].

In contrast, ectopic expression of Kv10.1 is frequently detected in tumor biopsies (Figure 1, Table 1) and cancer cell lines; several reports estimate that Kv10.1 is overexpressed in around 70% of clinical tumor samples [24,41,42,43,44,45,46,47,48], where the Kv10.1 expression seems to confer some malignant or oncogenic properties, e.g., sustained proliferation. This effect has been corroborated by in-vitro and in-vivo pharmacological studies using non-selective Kv10.1-blockers or siRNA, both showing positive results (Table 2), i.e., reducing proliferation rate in cancer cell lines or xenograft tumor size in immunodeficient mice [24,31,32,49,50,51,52].

Besides, Kv10.1 ectopic expression has been linked to chemoresistance cell phenotypes. Thus, Kv10.1 channel inhibition could improve cell response to therapeutic drugs typically used in chemotherapy. In-vitro experiments in ovarian cancer cells showed that Kv10.1 expression is associated with chemoresistance to cisplatin. The combination of Kv10.1 downregulation and cisplatin increased cell apoptosis in ovarian cancer cells compared with cells treated with cisplatin alone [53]. Similar results have been observed in the chemoresistant glioblastoma cell line U251AR, a cell line that showed high Kv10.1 (mRNA and protein) levels. Interestingly, U251AR cells became more sensitive to chemotherapeutic drugs when Kv10.1 expression was downregulated [54]. Agarwal et al. showed apoptosis induction by chemotherapeutic drugs (cytarabine, etoposide, idarubicin, and doxorubicin), commonly used to treat acute myeloid leukemia (AML), induced some degree of caspase activation in PLB-985 and K562 cells and primary cultured cells from a patient with AML. However, when these drugs were combined with astemizole or mAb56 (Kv10.1 blockers), this resulted in a further increase of caspase activity and, therefore, higher apoptosis, which is a desirable therapeutic effect [44].

On the other hand, radiotherapy has been widely used for several years to treat cancer in many ways and with high efficiency. To assess cellular radioresistance, Luis et al. irradiated with gamma radiation (4 Gy) to HEK293-WT cells and HEK293 cells stably expressing the Kv10.1 channel. Survival curves showed HEK-Kv10.1 cells remained unchanged after irradiation. In contrast, the survival curve of HEK-WT cells dramatically decreased with a 4 Gy radiation, suggesting that the expression of Kv10.1 confers to cells a radioresistant phenotype [55].

**Table 1:** Kv10.1 expression in cancer.

Cancer Type/Sample	Technique	Number of Samples	Kv10.1 Level Expression in Tumor Tissue	Kv10.1 Level Expression in Normal Tissue	Ref.
Liver carcinoma (hepatocellular carcinoma PTB)	IHC/RT-PCR	10	100% strongly positive	Completely negative	[41]
Colon cancer (colonic crypt cells)	Reverse transcription-PCR/FISH	2	100% detected	No transcripts were detected	[57]
Cervical cancer (primary culture biopsies)	Reverse transcription-PCR/SB	6	100% detected	33% detected in normal biopsies (*n* = 12)	[58]
Lung cancer (bronchus carcinoma PTB)	IHC/RT-PCR	10	90% highly positive	Bronchial epithelium was negative; some Kv10.1 expression in the sub mucous glands	[41]
Prostate cancer (Prostate Carcinoma)	IHC/RT-PCR	56	98% strongly positive	Faintly detectable in normal prostate epithelium	[41]
Cervical cancer (DCC)	ICC	13 HG; 36 LG	92% in HG-DCC; 67% in LG-DCC	27% of the normal samples(*n* = 184)	[59]
Prostate cancer(tumor tissue)	RT-PCR/IHC	-	89% of ADPCa 87% of AIPCa	7% in normal peritumoral tissue of ADPCa and AIPCa	[60]
Breast cancer (breast carcinoma PTB)	IHC/RT-PCR	230	Detected in 85%	The mammary epithelium was negative	[41]
Cerebral cancer (brain metastases, GBM)	IHC	75 BM; 71 GBM	85% of brain metastases. 78% of GBM	Under physiological conditions, Kv10.1 expression is restricted to the CNS	[61]
Head and neck cancer (HNSCC biopsies)	Reverse transcription-RT-PCR	54	83% of HNSCC. 39% in normal adjacent epithelium of HNSCC	No expression was detected in normal epithelia from non-oncologic patient (*n* = 44)	[47]
Colon cancer (colon carcinoma) PTB	IHC/RT-PCR	8	75% strongly positive	Colon epithelium was negative or slightly positive	[41]
Sarcoma (soft tissue biopsies)	IHC	210	71% of soft tissue sarcoma(82% rhabdomyosarcoma; 75% synovial sarcoma)	Negative in surrounding normal tissue	[42]
Acute myeloid leukemia (blood or bone marrow)	RT-PCR	118	40% of AML samples (56% and 60% in M2 and M6 subtypes, respectively)	Not detectable in healthy peripheral blood cells (*n* = 10)	[44]
Ovarian cancer (biopsies)	TMA	336	16% high expression. 58% low/intermediate expression	Low level expression in normal ovary (*n* = 6)	[43]

Acute myeloid leukemia (AML), androgen-dependent prostate cancer (ADPCa), androgen-independent prostate cancer (AIPCa). Brain metastasis (BM), cancer tissue microarray (TMA), dysplastic cervical cytologies (DCC), fluorescence in-situ hybridization (FISH), glioblastoma multiforme (GBM), head and neck squamous cell carcinoma (HNSCC), high grade (HG), immunocytochemistry (ICC), immunohistochemistry (IHC), low grade (LG), real-time polymerase chain reaction (RT-PCR), Southern-blot (SB), primary tumor biopsies (PTB).

**Table 2:** Kv10.1 effects on cancer cell lines and/or its pharmacological inhibition.

Cancer Type (Cell Line)	Cell Process	Technique	Kv10.1 Participation in Cell Process	Kv10.1 Inhibition	Ref.
Breast cancer (MCF7)	Cell Proliferation	Pharmacological inhibition/MTS/flow cytometry	IGF-1 increased mRNA expression of Kv10.1 in a time-dependent manner with an enhancement of cell proliferation	Astemizole (10 µM) and Quinidine (20 µM) inhibited cell proliferation induced by IGF-1	[62]
Breast cancer (MCF7)	Cell Proliferation/Cell cycle	Electrophysiology/RT-PCR/Pharmacological inhibition/3H-Thymidine	Kv10.1 channel activity varied in a cell cycle-dependent manner	TEA (2, 6, and 10 mM) and Astemizole (2, 5, and 10 µM) decrease proliferation and accumulate cells in G1 phase of the cell cycle	[63]
Breast cancer (MDA-MB-231)	Cell Migration	Pharmacological inhibition/siRNA/wound healing assay	Kv10.1 is required for cell migration by regulating Ca2+ entry through Orai1 channels	Astemizole (5 µM) reduces cell migration (45%)	[64]
Breast cancer (MDA-MB-231)	Cell Migration	Patch-clam/Pharmacological inhibition/siRNA/wound healing assay.	---	Chloroquine (30 μM) inhibited 34% of potassium currents, and 100 μM decreased cell migration (38%)	[65]
Leukemia cell lines (PLB-985, UT-7, K562, HEL)	Cell proliferation	Pharmacological inhibition/siRNA	---	Astemizole (4 μM) and imipramine (20 μM) inhibited PLB-985, UT-7, and K562 cell proliferation (up tp 77%); Knockdown of Kv10.1 expression by siRNA in PLB-985 and K562 cells diminished up to 80% cell proliferation	[44]
HNSCC-derived cell line (SCC42B, SCC40)	Cell proliferation/Cell invasion	siRNA/MTS/Matrigel invasion assays	Involvement of histone acetylation (i.e., H3Ac and H4K16Ac activating marks) in the regulation of Kv10.1 expression in HNSCC	Kv10.1 inhibition by siRNA reduced cell proliferation as well as invasive cell capacity	[47]
Prostate cancer (RWPE-1, WPE1-NB26)	Cell proliferation	RT-PCR/Fluorescence	WPE1-NB26 cells express high Kv10.1 protein in contrast to RWPE-1 cells expression.	Astemizole (2 µM) decreased RWPE-1 cell proliferation; Astemizole (2 μM) induced apoptosis in the WPE1-NB26 cells	[49]
Soft tissue sarcoma cell lines (RMS: TE-671, A-204; FS: HT-1080, Hs633t)	Cell proliferation	Pharmacological inhibition/siRNA	Kv10.1 participates in the proliferation of soft tissue sarcoma cell lines	Imipramine (10 μM) and hEag1 inhibition by siRNA reduced cell proliferation (82%)	[42]
Malignant melanoma cells (IGR1)	Cell proliferation	Pharmacological inhibition/BrdU incorporation/MTT assay	Kv10.1 expression may be of importance for the proliferation of melanoma cells	Imipramine (10 μM) reduces IGR1 cells proliferation; 30 μM imipramine induces IGR1 cells apoptosis	[66]
Ovarian cancer (KKOV3 and TYK)	Cell apoptosis	Inhibition by siRNA/apoptosis assay/RT-PCR	Kv10.1 regulates cell apoptosis via NF-kB pathway. Kv10.1 regulates P-glycoprotein expression	Knockdown of Eag1 by siRNA facilitated the sensitivity of ovarian cancer cells to cisplatin-induced apoptosis	[53]
Human glioblastoma cells (U251 and U251AR)	Cell growth and multi-drug resistance	Inhibition by miRNA/RT-PCR/drug sensitivity assay	Kv10.1 is involved in multi-drug resistance in glioblastoma cells	miR-296-3p regulates negatively Kv10.1 and suppresses cell proliferation drug resistance	[54]

Fibrosarcoma (FS), head and neck squamous cell carcinoma (HNSCC), microRNA (miRNA), nuclear factor k-light chain-enhancer of activated B cells (NF-kB), real-time polymerase chain reaction (RT-PCR), rhabdomyosarcoma (RMS), small interfering RNA (siRNA), tetraethylammonium (TEA).

The Kv10.1 enriched expression in cancer cells, its limited expression in other tissues and its membrane localization make Kv10.1 a promising protein in cancer research, becoming a potential tumor marker or an anti-cancer drug target, with special interest on the molecular mechanisms that regulate its expression.

#### 2.4.1. Regulatory Mechanisms of Kv10.1 in Cancer

Kv10.1 is under direct control of p53 and the retinoblastoma protein (pRb), two of the most altered proteins in cancer [67]. When p53 and pRb are down-regulated in cancer, the repression of the transcription factor E2F1 is lost [68]. Because in the promotor region of the *KCNH1* gene there exist E2F1-responsive elements, up-regulation of E2F1 increases the Kv10.1 levels and vice versa [40,68].

Similarly, it has been shown that the human papillomavirus (VPH), considered one of the leading agents associated with cervical cancer, contains the transforming viral proteins E6 and E7, which are associated with enhancing proliferation and cancer progression [69]. These two oncoproteins, E6 and E7, target and reduce the expression of p53 and pRb, respectively, thereby increasing E2F1 and Kv10.1 expression. A positive correlation between E7 and Kv10.1 has been observed in cervical cancer biopsies [58].

Likewise, microRNAs (miRNAs) could control the expression of protein-coding genes, including ion channels [70,71,72]. In this context, p53 reduction would cause a decrease in miR-34a, which may function as a translational inhibitor of Kv10.1. miR-34a has a dual mechanism of Kv10.1 inhibition, a direct way at post-transcriptional level and an indirect way at the transcriptional level through repressing E2F1 [68]. Also, it has been reported that miR-296-3p is a Kv10.1-modulator. In glioblastoma, down-regulation of miR-296-3p increased Kv10.1expression [54]; interestingly, miRNA’s expression could be associated with multi-drug resistance, probably through regulating Kv10.1 [54]. Similarly, it has been observed that Kv10.1 expression seems to confer a radioresistant phenotype [55].

As we mentioned above, Kv10.1 expression could be associated with hormonal factors. In HeLa cells and cervical cancer primary cultures, estrogens have been observed to up-regulate Kv10.1 [73]. Besides, insulin-like growth factor-1 (IGF1) is involved in tumor overexpression of Kv10.1, an effect mediated by the Akt-dependent pathway [62]. In contrast, calcitriol, the hormonally-active form of vitamin D3, decreases Kv10.1 expression [50] through a negative vitamin D-response element located in the *KCNH1* promoter [74].

#### 2.4.2. Epigenetic Regulation

Epigenetic changes are well-recognized mediators in gene expression and one source of variation in cancer. Last year, significant advances were made in understanding cancer epigenetics, especially regarding aberrant DNA methylation, chromatin remodeling, histone acetylation and methylation, posttranslational modifications, and non-coding RNAs deregulation [75].

DNA methylation involves the transference of a methyl group to the 5-carbon on cytosine residues in CpG sites [75]. A study on DNA methylation in human gastric carcinoma found hypermethylation in the CpG island of 15 genes, *KCNH1* was among them, suggesting a significant role in gastric cancer development [76]. *KCNH1* showed a three-times methylation-positive rate in gastric carcinoma samples than in noncancerous control samples [76]. However, it is needed to associate whether *KCNH1* hypermethylation may affect its expression in gastric carcinogenesis.

It has been proposed that early-life epigenetic changes may modify lung cell function and contribute to asthma risk [77]. Recent work found that early-life exposure to house dust mite allergen in a mouse model can alter DNA methylation and expression of diverse genes detected until three successive generations and associated with airway hyperresponsiveness and inflammation [77]. In the mice exposed to the allergens, the *Kcnh1* gene was hydroxymethylated and up-regulated, suggesting an association with asthma susceptibility [77].

In the head and neck squamous cell carcinoma (HNSCC), histone acetylation, but not DNA methylation, was suggested to be involved in the Kv10.1 regulation [47]. This work observed enrichment of two histone activating marks, H3Ac (H3 acetylation) and H4K16Ac (H4 lysine 16 acetylation), in HNSCC-derived cell lines expressing Kv10.1 [47].

MiRNAs are non-coding RNAs that regulate gene expression. As mentioned above, Kv10.1 can be modulated by diverse miRNAs that favor or repress its expression (Table 2) [54,68].

### 2.5. Kv10.1 Protein Degradation

Ion channels are highly fine-tuned proteins that are essential for cell physiology. Thus, the synthesis and degradation of ion channels should be a quality control process, which is necessary for correct cell function [78]. In this way, the ubiquitin system is the most studied mechanism of ion channel regulation. Nevertheless, knowledge about Kv10.1 synthesis and degradation is limited. Recently, it has been described that Kv10.1 protein degradation is dependent on E3 ubiquitin ligase cullin 7 (Cul7). Cul7 degrades both endoplasmic reticulum-localized immature and plasma membrane-localized matures Kv10.1 protein through the proteasomal and the lysosomal pathway, respectively [79]. In cooperation with this mechanism, the E3 ubiquitin ligase makorin ring finger protein 1 (MKRN1) has been described as another endoplasmic reticulum-localized mechanism of Kv10.1 degradation [80]. Both works demonstrated that Cul7 and MKRN1 are quality checkpoints of immature Kv10.1 channels.

## 3. Genetic Profile of *KCNH1* Gene Using cBioPortal

Genomic profiling of tumor samples is a valuable tool to unravel potential tumor biomarkers. Genomic studies from cohort studies may provide researchers with insights into the more advanced genomic landscape of cancer pathogenesis and treatment. Nevertheless, incomplete information about the epidemiology and clinical profile of cancer cases has been a major obstacle to complex genomic exploration. In the last few years, open-access resources have been made available to datasets for cancer exploration.

The public cBioPortal for cancer genomics (http://cbioportal.org, accessed on 24 July 2022) is an open-access resource for interactive exploration of multidimensional genomic datasets hosted by the Center for Molecular Oncology at MSK [81,82].

**Figure 2:**
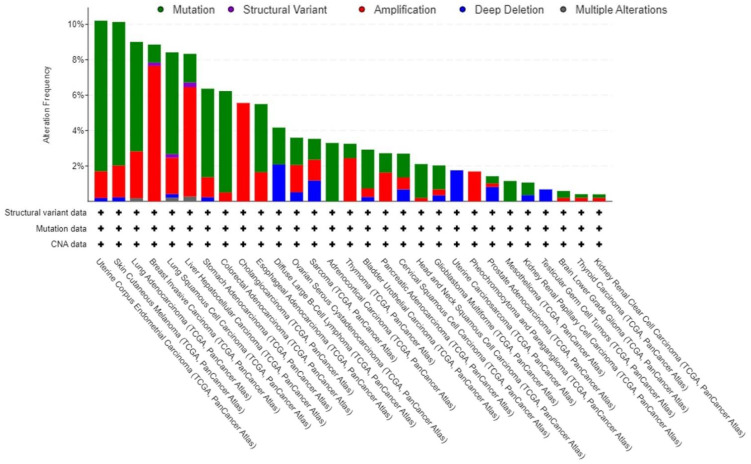
Genetic alteration profile of *KCNH1* gene using cBioPortal. Summary of genomic alteration frequency of *KCNH1* gene in different types of cancer (TCGA, PanCancer Atlas Studies). Copy-number alterations (CNA).

In this review, the cBioPortal database was used to query the *KCNH1* gene in TCGA PanCancer Atlas Studies (10,953 patients/10,967 samples). As shown in Figure 2, in distinct types of cancer, *KCNH1* undergoes different genetic alterations, amplification being the most frequent in breast cancer. Therefore, this cancer is explored below.

Breast cancer represents the most common cancer for women worldwide, with an estimated 47.8% of cancer incidence and 13.6% of cancer mortality [83] (GLOBOCAN 2018). Even though the molecular mechanisms associated with breast tumorigenesis have been extensively studied, the prognosis for different types is still unsatisfactory, indicating the necessity to find new biomarkers contributing to clinical decision-makers.

**Figure 3:**
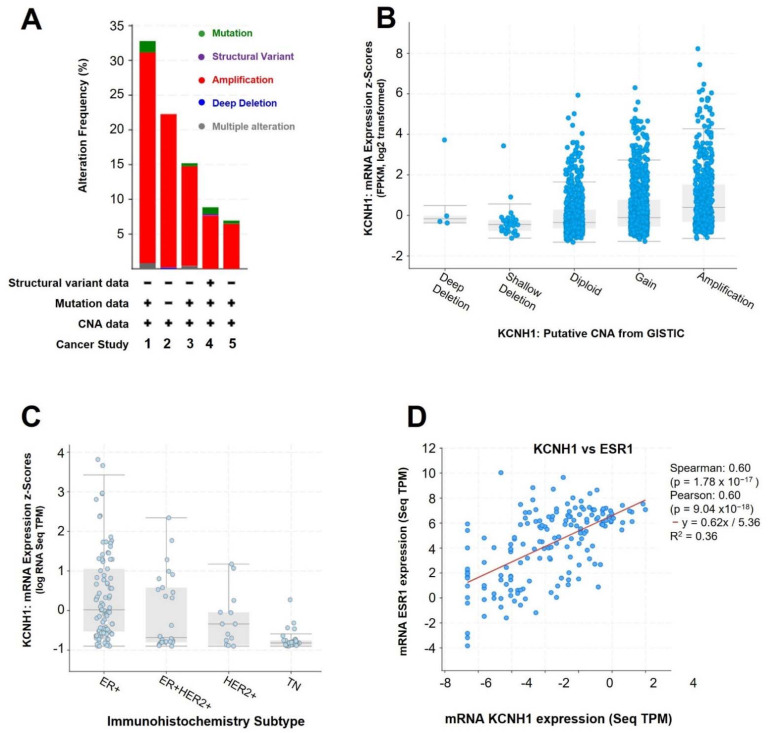
Genetic profile of *KCNH1* gene in breast cancer using cBioPortal. (**A**) Summary of genomic alteration frequency of *KCNH1* gene in five studies (1: CPTAC, Cell 2020; 2: METABRIC, Nature 2012 & Nat. Commun. 2016; 3: Provisional, February 2020; 4: TCGA, PanCancer Atlas; 5: INSERM, PLoS Med 2016). (**B**) Relative expression levels as a function of relative copy number of *KCNH1* gene (Breast invasive carcinoma, TCGA, PanCancer Atlas). (**C**) Relative expression levels as a function of the immunohistochemistry subtype of breast cancer (Breast Cancer, SMC 2018). (**D**) Copy-number alterations (CNA), fragments per kilobase of transcript per million fragments mapped (FPKM), genomic identification of significant targets in cancer (GISTIC), sequencing transcript per million (Seq TPM), variants of uncertain significance (VUS).

Gene amplification is a mechanism of deregulation of oncogenic activation, which results in overexpression of a gene and enhanced levels of the specific product. The amplification of the *KCNH1* gene is reported in five breast cancer studies, with a variation between 6.5–30.3% (Figure 3A). Because of this, gene amplification may have some clinical implications like diagnostic, prognostic, and predictive biomarkers in cancer [84]. For example, *ERBB2* (*HER2*) gene amplification is a molecular target in breast cancer therapy [85,86].

The breast cancer (TCGA, PanCancer) dataset was selected to observe the relationship between copy number alterations and mRNA levels (Figure 3B). The gene amplification in low-level (gain) and high-level (amplification) show the highest levels of mRNA *KCNH1* expression, which may account for the increased expression of *KCNH1* in breast cancer [41].

Finally, the breast cancer (Breast Cancer, SMC 2018) dataset was chosen to study the relationship between RNA levels and immunohistochemistry subtype. Finally, the breast cancer (Breast Cancer, SMC 2018) dataset was chosen to study the relationship between RNA levels and immunohistochemistry subtype and its co-expression with other genes. Breast cancer samples with ER+ subtype showed significantly higher mRNA *KCNH1* expression (Figure 3C). *KCNH1* upregulation can be explained by the co-expression of the estrogen receptor (*ESR1*) and *KCNH1* genes (Figure 3D). Experimental studies already have reported that estrogens can regulate the expression of *KCNH1* [72]. Furthermore, other genes such as *ABAT, CELSR1, FOXA1, HHAT, CCDC96, RUNDC1, ELOVL5, CAPN8, LRRC46,* and *ZMYND10* show high co-expression with *KCNH1* (Spearman correlation 0.68–0.71; *p* < 1.0 × 10^−23^). Among them, the *CAPN8* and *LRRC46* genes have been poorly studied in breast cancer.

## 4. Hallmarks of Cancer and Kv10.1 Expression

An emerging research area is concerned with the role of ion channel expression and its implication beyond their classical functions as action potential generators or players in neuronal communication [53,54]. In this way, numerous reports have shown that ectopic ion channel’s expression impacts cancer’s hallmarks and thus the disease progression [8].

The oncogenic properties of Kv10.1 were first observed when its over-expression in heterologous systems led to a phenotypic transformation compatible with cancer cells, which are characterized by faster growth rates (even at low serum concentrations), loss of contact inhibition, and solid tumors formation when these cells were transplanted into immunodeficient mice [24]; this work detonates the studies of Kv10.1 in cancer biology; however, not all its properties are completely understood.

### 4.1. Proliferation and Cell Cycle

Ectopic expression of Kv10.1 is strongly associated with an increase in the rate of cell proliferation, which may be due to the simultaneous activation of several signaling pathways. One of them is related to its canonical function as an ion channel: effluxing K^+^, hyperpolarizing the membrane potential, and increasing the driving force for Ca^2+^ entry to the cell; once inside of the cell, Ca^2+^ would start different cascade signaling, including cell proliferation [87,88].

In this regard, changes in the expression and activity of ion channels during all cell cycle phases have been described [87,88], generating crucial cyclic oscillations in the membrane potential. Depolarization of the membrane potential seems essential in the early G1 phase, and hyperpolarization appears important for the transition from G1 to S phase during mitosis [87,88]. Similar observations were described by C.D. Cone, where the membrane potential was proposed as a cell cycle regulator, shows that fast proliferating cells (e.g., cancer cells) are more depolarized, while cells with hyperpolarized membrane potential (e.g., neurons) show no mitotic activity [89].

As we have seen, Kv10.1 transcription is regulated by factors implicated in cell cycle progression (most of them converging in E2F1); moreover, Kv10.1-pharmacological inhibition reduces cell proliferation rate in cells where it is overexpressed, suggesting Kv10.1 is temporally regulated. The first indication of this phenomenon was that Kv10.1-permeability and -expression level depends on the cell cycle [55]. Recently, it was demonstrated that E2F1, which controls Kv10.1, would be expressed just during a short period in the cell cycle and not constitutively as had been thought for many years. Thus, Kv10.1 is expressed preferentially in the transition G2/M in cancer and normal cells, and when Kv10.1 is down-regulated, the G2/M transition is delayed, and cells take longer to complete mitosis [40].

### 4.2. Primary Cilium: Proliferation and Migration through Microtubule Dynamics Modulation

The primary cilium is a non-motile microtubule-based organelle similar to an antenna that extends outside the cell surface and is essential for the transduction of different signals implicated in proliferation, differentiation, migration, etc. [90]. Since the Kv10.1 is known to have a premitotic expression during the transition G2/M, some authors suggest that the strong implications of the Kv10.1 channel in cell cycle progression are also related with cilia disassembly that typically provides signaling needed to start proliferation [35,40]. Its participation in that process is evident since the cyclic expression of Kv10.1 has been shown to be present in the base of primary cilia of hTERT RPE-1 cells, which might result in a significant disposability of microtubules for consequent processes [91]. This signaling occurs soon after the Kv10.1-induced hyperpolarization favors the increase of Ca^2+^ moving through different calcium-permeable ion channels (L-Type, TRPC1, TRPV4, TRPP1, TRPP2, PKD2L1) [92,93,94]. Consequently, the increase of cytosolic Ca^+2^ recruits CaM activation. As a result, CaM triggers Aurora Kinase A (AURKA) activation, a serine-threonine protein kinase displayed to act as an effector protein in primary cilia reabsorption [92]. Otherwise, according to an AURKA interactome inspired by The Cancer Genome Atlas Program (TCGA), it is reported that AURKA can establish interactions between a large number of proteins involved in different oncogenic-related pathways such as FOXO, PI3K-AKt, Hippo signaling pathway, and apoptosis [95].

In regards to microtubule activity, the upper regulation of Kv10.1 is closely implicated in a significant increase of microtubule dynamics characterized by the growth rate of assembly and disassembly [96], because of this, an important linkage between Kv10.1 overexpression and some processes that include organelles and cell motility exists. Therefore, this is likely to connect the microtubule dynamics with the activation of Rho/GTPase, which is highly involved in actin polymerization led by myosin. Those cytoskeletal elements provide the required heading structures involved in focal adhesions, which are tightly related with cell migration owing to its interactions with the extracellular matrix [97]. However, the link between the Ca^+2^ channel PIEZO2 and the Rho activation has just been experimentally demonstrated [98]. Furthermore, a checkpoint Kv10.1-dependent named spindle assembly checkpoint (SAC) has been displayed, which is consistent with the fact that kinetochore-microtubule binding during cell division showed a decrease in error alignment rate of 20% and generates a delay before anaphase starting; these dependent Kv10.1 responses are suggested to be engaged with the activity of mitotic checkpoint complex (MCC) [96].

Moreover, a significant decrease in MDA-MB-231 cell migration rate when Kv10.1 is exposed to specific blockers like astemizole has already been proved [64]. Some hypotheses concluded that cell migration led by Kv10.1 channel could mainly trigger changes in microtubule dynamics as a result of hyperpolarization of action potential, which have been proved to induce calcium entry through ORAI1, and their association with secretory pathway Ca^2+^ ATPase (SPCA2) seems to be also implicated in proliferation and survival in cancer cells [99]. In contrast, it is thought that the interactions between Kv10.1 and cell migration are mediated mainly by the phosphorylation of kinase focal adhesions (FAK) [100].

### 4.3. Non-Canonical Role of Kv10.1 as Ion Channel and Angiogenesis

Ion channels can influence different signaling pathways through non-canonical function, involving protein-protein interactions independent of ion flux [101,102]. It might be surprising that some functions of Kv10.1 on cancer progression would be separate from its conducting properties. Transfection of the *Drosophila* Kv10.1 channel into mammalian cultured cells stimulated cell proliferation. This effect is maintained even when non-conducting channel mutants were used through an increase in the p38 MAPK activity [103].

It has been shown that the non-conducting properties of Kv10.1 seem to give rise to tumors and angiogenesis. Transplant of cells expressing non-conducting Kv10.1 mutants can produce tumors, indicating ion permeation is not required [52]. The same study showed that conductive and non-conductive Kv10.1 channels favor neovascularization in tumors, a mechanism mediated by the up-regulation of the hypoxia-inducible factor 1α (HIF-1α) and the vascular endothelial growth factor (VEGF) [52].

### 4.4. Cell Survival

Also, trial cooperation between the Ca^2+^ channel Orai1, SPCA2 protein, and Kv10.1 has been recognized with strong participation in intracellular Ca^2+^ entry but also plays a significant role in collagen-1-induced breast cancer cell survival via DDR (Discoidin-Domain Receptors), a specific collagen 1 receptor, that seems to be tightly involved in ERK1/2 phosphorylation [104]. Cell survival via Orai1, SPCA2, and Kv10.1 may be explained by protein-protein interactions between each element implicated. For instance, the N-terminal and C-terminal of SPCA2 interact with Kv10.1. The former terminal seems to participate in Kv10.1 expression, trafficking, and activity, while the C-terminal interactions evoke trafficking, expression, activity, and Ca^2+^ entry [99]. That partnership cooperation was demonstrated to promote the Ca^2+^ entry just after the Kv10.1 membrane hyperpolarization occurs [105].

### 4.5. Cell Metabolism Remodeling

In 2011, Hanahan & Weinberg provided a new generation of cancer hallmarks by introducing emergent hallmarks just as (1) avoiding immune destruction and (2) cell metabolism remodeling [106]. On the latter, overwhelming results show that Kv10.1 is also responsible for metabolism remodeling in cancer cells through mitochondrial metabolism modulation. The Kv10.1 dependent-mitochondrial metabolism was tested by measuring whether mitochondrial metabolism inhibitors, such as Metformin, Phenformin & VLX600 had a differential effect over cell lines with a moderate expression of Kv10.1 in contrast to cells with Kv10.1 overexpressed. The results showed that in HeLa, Du145, and MDAMB43S5 cells, where Kv10.1 was overexpressed, the metabolism inhibitors’ sensitivity was increased, while in those cells with Kv10.1 moderate expression (MCF-7, MDAMB231, MiaPaCa2 & Panc-1) a poor metabolism inhibitor sensitivity was shown. In the same way, this could explain how metabolism Kv10.1-dependence is involved in apoptosis inhibition, since when Kv10.1 is blocked or its expression is inhibited, it triggers a mitochondrial fragmentation that leads to an increase of reactive oxygen species (ROS) that ultimately promotes increased autophagy [107].

## 5. Tumor Microenvironment and Kv10.1

The tumor microenvironment (TME) is a complex and continuously evolving entity. This microenvironment is generated by several factors such as low extracellular pH, hypoxic regions, metabolic abnormalities, and extracellular matrix modifications [108].

One distinguishing feature is that cancer cells develop in acidic microenvironments, small regions where the extracellular pH (pHe) is low compared with normal cells [109]. Under physiological conditions, extracellular pH in most of the body’s tissues is around 7.3–7.4, while in microenvironments created by tumors, pHe can drop to ranges between 6.2 and 6.8 [109,110]. It has been observed that extracellular pH acidification inhibits voltage-gated K^+^ channels [111], including Kv10.1 [112,113]. This inhibition could explain, in part, the overexpression of this channel in cancer cells as a compensatory mechanism. Nevertheless, in T_84_ colonic carcinoma cells, hyperpolarization of the membrane potential induced by Kv10.1 (and other Kv channels) controls cell proliferation by affecting intracellular pH and Ca^2+^ signaling [114].

The extracellular matrix (ECM) is a 3D structure of different protein components, including collagen, fibronectin, proteoglycans, and glycoproteins [108]. During the carcinogenic process, changes in the TME induce alterations in the ECM components that contribute to the tumor progression. Indeed, it has been observed that ECM components modulate Kv10.1. CHO cells expressing Kv10.1 suffer a cytoskeleton and lamellipodia reorganization when grown on collagen, suggesting an increase in cell movement [115]. In contrast, growing cells on fibronectin induce the formation of stress fibers, indicating a role of Kv10.1 in the cytoskeletal organization in cancer [115]. Furthermore, compared with CHO-WT cells, those cells expressing the Kv10.1 channel could proliferate despite being kept under total serum deprivation [115]. Interestingly, it has been observed that collagen 1 increases cell survival in breast cancer cell through ERK1/2 phosphorylation and the overexpression of Kv10.1 and ORAI1, which are effects mediated by the activation of the tyrosine kinase receptor activated by collagen, DDR1 [104].

On the other hand, a relationship has been observed between Kv10.1 expression and an increase in vascular endothelial growth factor (VEGF) secretion, as well as the expression of hypoxia-inducible factor 1α (HIF-1α), one of the most important factors promoting angiogenesis. It has been proposed that Kv10.1 interferes with hypoxia homeostasis by increasing basal HIF-1α activity and lowering the threshold of HIF-1α activation [52]. Besides, Lai et al. showed that co-expression of the Kv10.1 channel and HIF-1α in human breast cancer samples were related to the tumor size and status [116].

## 6. Conclusions

For over 70 years, knowledge about ion channels has increased exponentially. Today, it is well known that ion channels are “probably” involved in all physiological processes, and their disfunction is the origin of diverse pathologies, making them pharmacological targets for the potential development of new compounds with clinical relevance. Currently, it is estimated that 18% of drugs used in the clinic have an ion channel as their target [117]. Drugs targeting K^+^ channels are used to treat metabolic, neurologic, and cardiac disorders with promising results [118].

**Figure 4:**
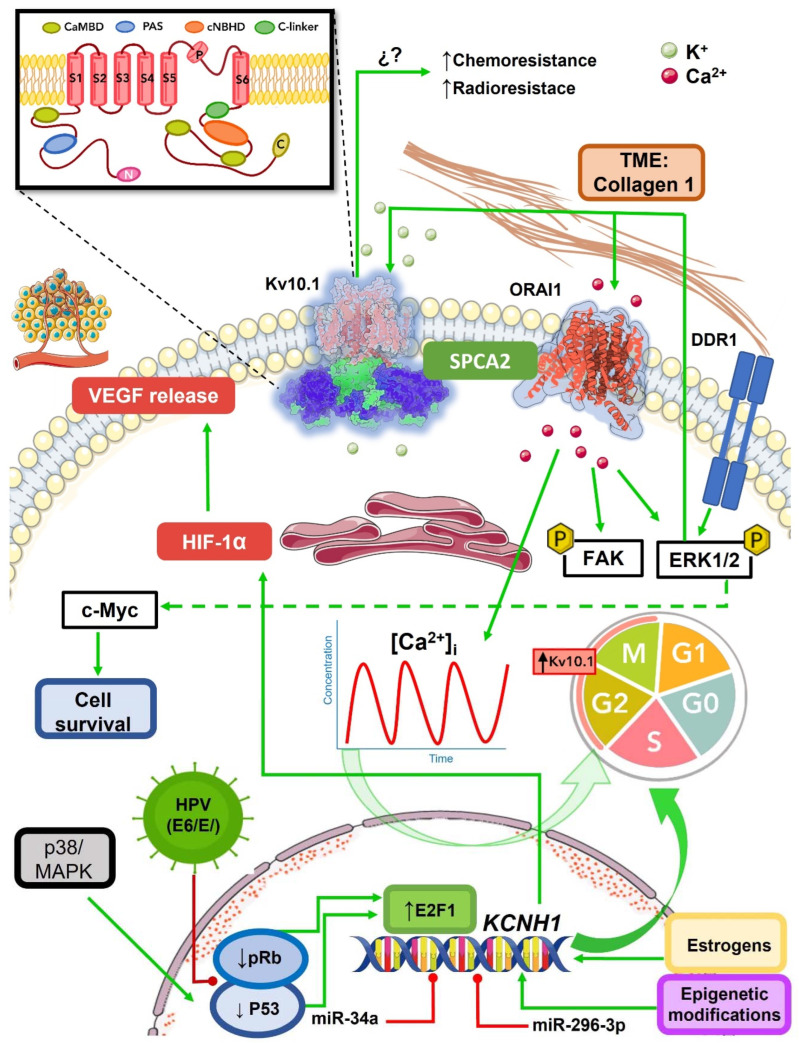
Schematic representation of Kv10.1 regulation in cancer. Kv10.1 is an ion channel permeable to K^+^ (green spheres) mainly localized in the plasma membrane, but also is found in subcellular structures. The crystal structure of the Kv10.1 channel is embedded in the plasma membrane, with the transmembrane region in red and the intracellular N- and C-termini represented in purple and green, respectively. The figure in the upper left corner represents a Kv10.1 subunit; transmembrane segments (S1–S6) are represented in red, the N-termini PAS domain in blue, the C-linker in green, the cNBDH in orange, and the 3 CaMBD in olive. A functional channel is assembled as a tetramer. Kv10.1 has been described as an oncogenic channel, exerting its effects through different hypothesized mechanisms. The canonical function of Kv10.1 consists of efflux K^+^ and hyperpolarized membrane potential. The membrane hyperpolarization increases the driving force for Ca^2+^, promoting the signal pathways involved in cell division. Kv10.1 expression is under the control of p53 and pRb; when p53 and pRb are down-regulated in cancer, an increase of E2F1 occurs, which triggers Kv10.1 expression. During the cell cycle, Kv10.1 is expressed during the G2/M transition. Alterations in the p53 and pRb pathways consequently affect the Kv10.1 levels, e.g., the oncoproteins E6 and E7 of the HPV reduce the expression of p53 and pRb, respectively. Other factors influencing Kv10.1 expression include hormones, miRNAs, epigenetic modifications, and components of the tumor microenvironment (e.g., collagen). Collagen 1 can activate the DDR1 pathway, increasing the ERK1/2 phosphorylation, which increases the Kv10.1 and ORAI1 levels in the plasma membrane. ERK1/2 phosphorylation, Kv10.1, and ORAI1 increased cell survival. Kv10.1 expression is also associated with angiogenesis through upregulation of the HIF-1α activity, increasing VEGF release. In addition, cells over-expressing Kv10.1 channels seem more resistant to cancer drug treatments and radiotherapy; however, the molecular mechanisms implicated in these resistant phenotypes are not completely understood.

Undoubtedly, the Kv10.1 channel plays an active role in carcinogenesis (Figure 4), becoming a protein with great potential for cancer therapeutics. Its membrane nature simplifies the development and carry-on of a molecule with pharmacological activity. As we have seen, its properties as a membrane protein, its high expression in cancerous tissue versus low expression in healthy tissue, and its participation in various cancer hallmarks make it a promising target for the design of new therapeutic strategies that combined with the current ones could decrease the tumorigenic process.

## Data Availability

Data from Section 3 is available in http://cbioportal.org.
